# Supplementary material for: Brucella Genomics: Macro and Micro Evolution
Source: Int J Mol Sci. 2020 Oct 20;21(20):7749. doi: 10.3390/ijms21207749 (PMC7589603; doi:10.3390/ijms21207749)
Supplement: Supplementary file 1 [file ijms-21-07749-s001.zip › MaterialSup/S1_Tables.docx]

**S1 Tables**.

Strains used for Alphaproteobacteria phylogenetic reconstruction.

| **Strain** |  | **Taxonomic Subgroup** |  | **Accession number** |
| --- | --- | --- | --- | --- |
|  |  |  |  |  |
| **OUTGROUP** |  |  |  |  |
| *Escherichia coli* K12 |  | Gammaproteobacteria |  | NC_000913 |
| *Geobacter sulfurreducens* PCA |  | Betaproteobacteria |  | NC_002939 |
| *Ralstonia solanacearum* GMI1000 |  | Deltaproteobacteria |  | NC_003295 |
|  |  |  |  |  |
| **RHIZOBIALES** |  |  |  |  |
| *Brucella abortus* 2308 Wisconsin |  | Brucellaceae |  | GCA_900095085.1 |
| *Brucella suis* str. 1330 |  | Brucellaceae |  | GCA_000007505.1 |
| *Ochrobactrum intermedium* LMG 3301 |  | Brucellaceae |  | GCA_000182645.1 |
| *Agrobacterium tumefaciens str. Ach 5* |  | Rhizobiaceae |  | NZ_CP011246.1 |
| *Sinorhizobium meliloti* str. RU11/01 |  | Rhizobiaceae |  | NZ_CP021219.1 |
| *Bradyrhizobium japonicum* str. J5 |  | Bradyrhizobiaceae |  | NZ_CP017637.1 |
| *Rodhopseudomonas palustris* TIE-1 |  | Bradyrhizobiaceae |  | NC_011004.1 |
| *Mesorhizobium loti* str. TONO |  | Phyllobacteriaceae |  | NZ_AP017605.1 |
| *Bartonella quintana* str. Toulouse |  | Bartonellaceae |  | NC_005955.1 |
| *Bartonella henselae* str. BM 1374165 |  | Bartonellaceae |  | NZ_HG969191.1 |
|  |  |  |  |  |
| **CAULOBACTERALES** |  |  |  |  |
| *Caulobacter crescentus* NA1000 |  | Caulobacteraceae |  | NC_011916.1 |
|  |  |  |  |  |
| **RICKETTSIALES** |  |  |  |  |
| *Rickettsia prowazeki* str. RpGvF24 |  | Rickettsiaceae |  | NC_017057.1 |
| *Rickettsia conorii* str. Malish 7 |  | Rickettsiaceae |  | NC_003103.1 |
| *Wolbachia pipientis* str. wPpe |  | Anaplasmataceae |  | GCA_001752665.1 |

Strains used for phylogenetic relationship of “classical” and “non-classical” *Brucella* species and *Ochrobactrum* sp.

| **Species and strains** | **Host specie** | **Common name** | **Accession number** |
| --- | --- | --- | --- |
| *Brucella* spp. BCCN 84-3 | *Canis familiaris* | Domestic dog | ERS568777 |
| *Brucella melitensis* 16M | *Capra aegagrus* | Goat | NC_003317.1 & NC_003318.1 |
| *Brucella neotomae* 5K/33 | *Neotoma lepida* | Wood rat | GCA_000158715.1 |
| *Brucella suis bv 1* st. 1330 | *Sus scrofa domesticus* | Domestic pig | NC_004310.3 & NC_004311.2 |
| *Brucella suis* st. PT09143 bv 2 | *Sus scrofa* | Wild boar | [GCA_000698245.1](file:///www.ncbi.nlm.nih.gov/assembly/GCA_000698245.1) |
| *Brucella suis* bv 2 str. ATCC 23445 | *Sus scrofa* | Wild boar | GCA_000018905.1 |
| *B. suis* bv 3 str. 686 | Not known | Not known | NZ_CP007719.1; NZ_CP007718.1 |
| *Brucella suis* st. F4/06-146 bv 4 | Not known | Not known | GCA_000365705.1 |
| *B. suis* bv 5 st. 513 | Not known | Not known | GCA_000157755.1 |
| *Brucella ceti* dolphin type TE10759-12 | *Stenella coeruleoalba* | Striped dolphin | CP006896 & CP006897 |
| *Brucella cet*i porpoise type F23-97 | *Tursiops truncatus* | Bottle nose dolphin | NZ_AQKR00000000.1 |
| *Brucella pinnipedialis* B2/94 | *Phoca vitulina* | Common seal | NZ_ACFF00000000 |
| *Brucella canis* ATCC 23365 | *Canis familiaris* | Domestic dog | NC_010103.1 & NC_010104.1 |
| *Brucella ovis* ATCC25840 | Not known | Not known | NC_009505.1 & NC_009504.1 |
| *Brucella microti* CCM 4915 | *Microtus arvalis* | Common vole | NC_013119.1 & NC_013118.1 |
| *Brucella* spp. F5-99 | *Tursiops truncatus* | Bottle nose dolphin | NZ_ACFF00000000 |
| *Brucella vulpis* F60 BVF60 | *Vulpes vulpes* | Red fox | GCA_900000005.1 |
| *Brucella inopinata* BO1 | *Homo sapiens* | Human | GCA_000182725.1 |
| *Brucella* spp. BO2 | *Homo sapiens* | Human | GCA_000177135.1 |
| *Brucella* spp. 09RB8910 | *Pyxicephalus edulis* | African bullfrog | NZ_CP019390 |
| *Brucella* spp. 10RB9215 | *Pyxicephalus edulis* | African bullfrog | LT599047 |
| *Brucella* spp. B13-0095 | *Ceratophrys ornata* | Pac man Frog | GCA_000157875.1 |
| *Brucella* spp. NF 2653 | *Melomys lutillus* | Small climbing rat | GCA_000177155.1 |
| *Brucella* spp. 141012304 | *Taenuria lymma* | Fantail ray | GCA_900095155.1 |
| *Brucella* *papionis* NVSL_07_0026 | *Papio* spp. | Baboon | GCA_000163135.1 |
| *Brucella* sp. 83/13 | Not known | Not known | [GCA_000157875.1](file:///www.ncbi.nlm.nih.gov/assembly/GCA_000157875.1) |
| *Brucella abortus* 9-941 | *Bos taurus* | Cow | GCA_000008145.1 |
| *Brucella* sp. 191011898 | *Furcifer pardialis* | Panther chameleon | PRJEB37990 |
| *Ochrobactrum intermedium* LMG3301 | Soil | Soil | NZ_ACQA00000000.1 |
| *Ochrobactrum anthropi* ATCC49188 | *Homo sapiens* | Human | NC_009667.1, NC_009668.1, NC_009669.1, NC_009670.1, NC_009671.1 & NC_009672.1 |
| *Ochrobactrum haematophilum* st. CCUG 38531 | *Homo sapiens* | Human | [GCA_005938105.1](file:///www.ncbi.nlm.nih.gov/assembly/GCA_005938105.1) |
