# Supplementary material for: Brucella Genomics: Macro and Micro Evolution
Source: Int J Mol Sci. 2020 Oct 20;21(20):7749. doi: 10.3390/ijms21207749 (PMC7589603; doi:10.3390/ijms21207749)
Supplement: Supplementary file 1 [file ijms-21-07749-s001.zip › MaterialSup/S3_Table.doc]

***S3 Table.*** *Genome characteristics of Brucella species that show genome reduction and increased pseudogenization.*

|  |  | ***B. ceti P1**** | |  | ***B. pinnipedialis*†** | |  | ***B. canis*†** | |  | ***B. suis*†** | |  | ***B. abortus*†** | |  | ***B. ovis*†** | |
| --- | --- | --- | --- | --- | --- | --- | --- | --- | --- | --- | --- | --- | --- | --- | --- | --- | --- | --- |
|  |  |  |  |  |  |  |  |  |  |  |  |  |  |  |  |  |  |  |
| **Feature** |  | **Chr I** | **Chr II** |  | **Chr I** | **Chr II** |  | **Chr I** | **Chr II** |  | **Chr I** | **Chr II** |  | **Chr I** | **Chr II** |  | **Chr I** | **Chr II** |
|  |  |  |  |  |  |  |  |  |  |  |  |  |  |  |  |  |  |  |
| Size (kb) |  | 2117 | 1179 |  | 2121 | 1236 |  | 2106 | 1207 |  | 2108 | 1207 |  | 2111 | 1164 |  | 2121 | 1157 |
|  |  |  |  |  |  |  |  |  |  |  |  |  |  |  |  |  |  |  |
| G-C content (%) |  | 57.14 | 57.26 |  | 56.96 | 57.03 |  | 57.21 | 57.21 |  | 57.3 | 57.3 |  | 57.2 | 57.3 |  | 57.2 | 57.2 |
|  |  |  |  |  |  |  |  |  |  |  |  |  |  |  |  |  |  |  |
| No. of CDS per chromosome |  | 1944 | 1026 |  | 2111 | 1171 |  | 2040 | 1118 |  | 2123 | 1149 |  | 2003 | 1045 |  | 1928 | 962 |
|  |  |  |  |  |  |  |  |  |  |  |  |  |  |  |  |  |  |  |
| No. of CDS per genome |  |  | 2970 |  |  | 3282 |  |  | 3158 |  |  | 3266 |  |  | 3048 |  |  | 2889 |
|  |  |  |  |  |  |  |  |  |  |  |  |  |  |  |  |  |  |  |
| No. of tRNAs per chromosome |  | 38 | 13 |  | 29 | 11 |  | 41 | 14 |  | 41 | 14 |  | 39 | 14 |  | 39 | 14 |
|  |  |  |  |  |  |  |  |  |  |  |  |  |  |  |  |  |  |  |
| No. tRNAs per genome |  |  | 51 |  |  | 40 |  |  | 55 |  |  | 55 |  |  | 53 |  |  | 53 |
|  |  |  |  |  |  |  |  |  |  |  |  |  |  |  |  |  |  |  |
| No. pseudogenes/ chromosome |  | 137 | 116 |  | 88 | 83 |  | 76 | 59 |  | 59 | 51 |  | 113 | 75 |  | 141 | 128 |
|  |  |  |  |  |  |  |  |  |  |  |  |  |  |  |  |  |  |  |
| No. pseudogenes per genome |  |  | 253 |  |  | 171 |  |  | 138 |  |  | 110 |  |  | 188 |  |  | 269 |
|  |  |  |  |  |  |  |  |  |  |  |  |  |  |  |  |  |  |  |
| IS*711* copies per genome |  |  | 47 |  |  | 46 |  |  | 7 |  |  | 8 |  |  | 6 |  |  | 33 |
|  |  |  |  |  |  |  |  |  |  |  |  |  |  |  |  |  |  |  |
| Pseudogene CDS fraction (*100) per chromosome |  | 7.05 | 11.31 |  | 4.17 | 7.09 |  | 3.72 | 5.27 |  | 2.78 | 4.44 |  | 5.64 | 7.18 |  | 7.31 | 13.31 |
|  |  |  |  |  |  |  |  |  |  |  |  |  |  |  |  |  |  |  |
| Pseudogene/CDS fraction (*100) per genome |  |  | 8.52 |  |  | 5.21 |  |  | 4.36 |  |  | 3.36 |  |  | 6.17 |  |  | 9.31 |

§ Pseudogene CDS fraction (*100) is calculated as follows: # pseudogenes/#CDS *100

* Genome assembly, numbers are approximate.

† Reference genome: *B. pinnipedialis* B2/94, *B. canis* ATCC 23365, *B. suis* st. 1330, *B. abortus* st. 9-941 and *B. ovis* ATCC 25840.

Modified from Suárez-Esquivel, et al., 2017a
