# Supplementary material for: Brucella Genomics: Macro and Micro Evolution
Source: Int J Mol Sci. 2020 Oct 20;21(20):7749. doi: 10.3390/ijms21207749 (PMC7589603; doi:10.3390/ijms21207749)
Supplement: Supplementary file 1 [file ijms-21-07749-s001.zip › MaterialSup/S4_Table.doc]

**S4 Table.** GIs reported in Brucella.

| **Island** | **Coordinates (locus tag)** | **Size (kb)** | **GC%** | **Site of integration** | **ORF content** | **Ref** |
| --- | --- | --- | --- | --- | --- | --- |
|  | | | | | | |
| **Chromosome I** | | | | | | |
|  | | | | | | |
| SAR1-17 | BMEI0051-0065 | 19.4 | 51.1 | tRNA_Val_ | 14 ORFs, adhesion proteins, exonuclease, metabolism | 1 |
| GI-1^Š^ | BMEI0801-0907 | 8.1 | 53.3 | tRNA_Leu_ | 9 ORFs, mostly PPs and phage-related proteins | 2 |
| GI-2^Š^ | BMEI0993-1012 | 15.1 | 51.3 | tRNA_Gly_ | 20 ORFs, LPS gly-transferases, omp25, PPs and phage-related | 2 |
| SAR1-5 | BMEI1218-1231 | 13.8 | 56.5 | tRNA_Arg_ | 13 ORFs, exonuclease, acetyltransferase, phage regulator, PPs | 1 |
| wbk | BMEI1393-1422 | 20.3 | 50 | tRNA_Gln_ | 26 ORF, LPS transport and O-PS synthesis. Several transposases | 1, 3 |
| SAR1-2 | BMEI1656-1664 | 8.1 | 53.7 | tRNA_Thr_ | 11 ORFs, PPs and phage-related genes | 1 |
| GI-3^¥^ | BMEI1674-1703 | 20.9 | 52.3 | tRNA_Phe_ | 30 ORFs, mostly PPs. TIR domain protein | 2 |
| GI-4 | BMEI1919-1923 | 5.2 | 56 | tRNA_Ala_ | 5 ORFs, acetoacetyl-CoA metabolism | 2 |
|  | | | | | | |
| **Chromosome II** | | | | | | |
|  | | | | | | |
| virB | BMEII0024-0034 | 12.8 | 56 | NI | 11 ORFs encoding T4SS required for virulence | 3 |
| GI-5^‡^ | BMEII0185-0226 | 44.1 | 57 | tRNA_Ser/Met_ | 42 ORF, ABC transporters, regulators, PPs, antibiotic activators | 2 |
| SAR2-10 | BMEII0447-0457 | 14.3 | 56.4 | tRNA_Thr_ | 13 ORFs, Restriction-modification system and PPs | 1 |
| GI-6 | BMEII0710-0719 | 7.5 | 54.7 | tRNA_Ser_ | 10 ORFs, transposases, transcription regulators, hemagglutinin | 2 |
| GI-7 | BMEII0811-0815 | 6.4 | 56 | NI | 5 ORFs, a PP, a transcriptional protein and metabolism | 2 |
| GI-8^§^ | BMEII0826-0850 | 25.1 | 58 | NI | 25 ORFs including omp31, sugar metabolism and LPS synthesis. | 2 |
| GI-9 | BMEII0875-0878 | 3.2 | 56 | NI | 4 ORFs, a transcription regulator, PP and metabolism | 2 |

| **Island** | **Coordinates (locus tag)** | **Size (kb)** | **GC%** | **Site of integration** | **ORF content** | **Ref** |
| --- | --- | --- | --- | --- | --- | --- |
|  |  | | | | | |
| 12Kb^†^ | BMI_II1048-1054 | 11.7 | 53.2 | tRNA_Leu_ | 5 ORFs, phage-related proteins | 3 |
| 26.5Kbˆ | BOV_A0483-0515 | 26.5 | 53.8 | NI | 32 ORFs, transposases, ABC transporter, haemagglutinin, PPs | 4 |
| 67kb^¦^ | BPI_II536 - 605 | 67.4 | 54 | ISBm1,  orfB | 57 ORFS, transposases, ABC transporters, PP, hemagglutinin, metabolism | 5 – 7 |
| GIBs2* | BR0584-0593 | 7 | 60.7 | NI | 10 ORF, mostly phage-related proteins | 3 |
| mtgC | BRA0036-0042 | 8 | 59.3 | tRNA_Val_ | 7 ORFs, Mg^2+^ uptake-related genes mgtB-mgtC and PPs | 8 |
| IncP^¶^ | BRA0361-0380 | 18.3 | 55.3 | NI | 18 plasmid-related ORFs | 9 |
| GIBs3* | BRA0630-0635 | 7.7 | 56.1 | NI | 6 ORF, ABC binding proteins, amino acid dehydrogenase, PP | 3 |
| 4Kb | BruAb2_0165-0171 | 10.1 | 56.2 | NI | 6 ORFs, PPs and an autotransporter | 3 |
| 13Kb | BruAb2_1085-1086 | 13.1 | 56.5 | NI | 2 ORFs, and PP an autotransporter | 3 |

**Exclusive of:** ^†^ *B. microti* | ˆ *B. ovis* | ¦ *B. pinnipedialis.* **ORF**, open reading frame; **NI**, not identified; **PP**, predicted protein.

**Absent in:** ^§^ *B. abortus* | ^‡^ *B. suis* | ^¥^ *B. canis*, *B. suis*, *B. microti* | ^Š^ *B.* ovis | * *B. melitensis* | ^¶^ *B. abortus*, *B .melitensis*, *B. ovis*.

**References: (1)** Wattam et al., 2009**, (2)** Rajashekara et al., 2004a**, (3)** Mancilla, 2012**, (4)** Tsolis et al., 2009, **(5)** Bourg et al., 2007**, (6)** Maquart et al., 2008**, (7)** Audic et al., 2011**, (8)** Blanc-Potard and Lafay, 2003**, (9)** Lavigne and Vergunst, 2005.
