# Supplementary material for: Brucella Genomics: Macro and Micro Evolution
Source: Int J Mol Sci. 2020 Oct 20;21(20):7749. doi: 10.3390/ijms21207749 (PMC7589603; doi:10.3390/ijms21207749)
Supplement: Supplementary file 1 [file ijms-21-07749-s001.zip › MaterialSup/S2_Table.doc]

**S2 Table.** Recognized and proposed *Brucella* species and strains.

| **Species** |  | **Preferential Host** |  | **Zoonotic Risk** |  | **Reference** |
| --- | --- | --- | --- | --- | --- | --- |
|  |  |  |  |  |  |  |
| *B. melitensis* |  | Sheep, goats |  | High |  | 1 |
| *B. abortus** |  | Bovine |  | High |  | 2, 3* |
| *B. suis* |  | Swine |  | High |  | 4 |
| *B. ovis* |  | Ovine |  | None |  | 5 |
| *B. neotomae** |  | Desert woodrat (*Neotoma lepida*) |  | High |  | 4, 6 |
| *B. canis** |  | Canines |  | Moderate |  | 7, 3* |
| *B. ceti** |  | Cetaceans |  | Unknown |  | 8 – 11* |
| *B. pinnipedialis* |  | Pinnipeds |  | Unknown |  | 9, 10 |
| *B. microti^1^* |  | Foxes, rodents^1^ |  | Unknown |  | 12 |
| *B. innopinata*^2,3^ |  | Unknown ^3^ |  | High |  | 13, 14 |
| *B. papionis*^2^ |  | Baboons |  | Unknown |  | 15 |
| Strain NF 2653^§a^ |  | Small climbing rat (*Melomys lutillus)* |  | Unknown |  | 16 |
| Strain B13-0095^§b^ |  | Pac-Man frog (*Ceratophyrus ornate*) |  | Unknown |  | 17, 18 |
| *B. vulpis*, strain F60 BVF60^§b^ |  | Red fox (*Vulpes vulpes)* |  | Unknown |  | 19 |
| Strain 141012304^§c^ |  | Ribbontail ray (*Taeniura lymma*) |  | Unknown |  | 20 |
| Strains 09RB8910^§c^ and 10RB9215^§c^ |  | African bullfrog (*Pyxicephalus edulis)* |  | Unknown |  | 21 |
| Strain BCCN84.3*^§d^ |  | Dogs |  | Unknown |  | 22* |
| Strain 191011898 |  | Chameleon (Furcifer pardalis) |  |  |  | 23 |

* Reported in CR; **^1^** *B. microti* has been isolated from ground; **^2^** *B. innopinata* and *B. papionis* are not recognized proposed species; **^3^** *B. innopinata* has been isolated from mammary implants and lung biopsies in humans, but no animal reservoir has been identified yet. ^§^ Reported atypical strains in ^a^2010, ^b^2016, ^c^2017, ^d^2019.

**References:**

1.	Bruce, D. Note on the discovery of a micro-organism in Malta fever. *Practitioner* **1887**, *39*, 161.

2.	Bang, B. Infectious abortion in cattle. *J Comp Pathol* **1906**, *19*, 191–202.

3.	Moreno, E. Brucellosis in Central America. *Vet. Microbiol.* **2002**, *90*, 31–38.

4.	Stoenner, H.G.; Lackman, D.B. A new species of *Brucella* isolated from the desert wood rat, Neotoma lepida Thomas. *Am J Vet Res* **1957**, *18*, 947–951.

5.	Buddle, M.B. Studies on *Brucella ovis* (n.sp.), a cause of genital disease of sheep in New Zealand and Australia. *J. Hyg.* **1956**, *54*, 351–364.

6.	Suárez-Esquivel, M.; Ruiz-Villalobos, N.; Jiménez-Rojas, C.; Barquero-Calvo, E.; Chacón-Díaz, C.; Víquez-Ruiz, E.; Rojas-Campos, N.; Baker, K.S.; Oviedo-Sánchez, G.; Amuy, E.; et al. *Brucella neotomae* Infection in Humans, Costa Rica. *Emerg. Infect. Dis. J.* **2017**, *23*, 997, doi:10.3201/eid2306.162018.

7.	Carmichael, L.E.; Bruner, D.W. Characteristics of a newly-recognized species of *Brucella* responsible for infectious canine abortions. *Cornell Vet* **1968**, *58*, 579–592.

8.	Ewalt, D.R.; Payeur, J.B.; Martin, B.M.; Cummins, D.R.; Miller, G.M. Characteristics of a *Brucella* species from a bottlenose dolphin (*Tursiops truncatus*). *J Vet Diagn Invest* **1994**, *6*.

9.	Ross, H.M.; Foster, G.; Reid, R.; Jahans, K.; MacMillan, A. *Brucella* species infection in sea-mammals. *Vet. Rec.* **1994**, *134*, 14.

10.	Foster, G.; Osterman, B.S.; Godfroid, J.; Jacques, I.; Cloeckert, A. *Brucella ceti* sp. nov. and *Brucella pinnipedialis* sp. nov. for *Brucella* strains with cetaceans and seals as their preferred hosts. *Int. J. Syst. Evol. Microbiol.* **2007**, *57*, 2688–2693, doi:10.1099/ijs.0.65269-0.

11.	Hernández-Mora, G.; González-Barrientos, R.; Morales, J.-A.; Chaves-Olarte, E.; Guzmán-Verri, C.; Barquero-Calvo, E.; Baquero-Calvo, E.; De-Miguel, M.-J.; Marín, C.-M.; Blasco, J.-M.; et al. Neurobrucellosis in stranded dolphins, Costa Rica. *Emerg. Infect. Dis.* **2008**, *14*, 1430–3, doi:10.3201/eid1409.071056.

12.	Scholz, H.C.; Hubalek, Z.; Sedláček, I.; Vergnaud, G.; Tomaso, H.; Al Dahouk, S.; Melzer, F.; Kämpfer, P.; Heubauer, H.; Cloeckaert, A.; et al. *Brucella microti* sp. nov., isolated from the common vole *Microtus arvalis*. *Int. J. Syst. Evol. Microbiol.* **2008**, *58*, 375–382, doi:10.1099/ijs.0.65356-0.

13.	De, B.K.; Stauffer, L.; Koylass, M.S.; Sharp, S.E.; Gee, J.E.; Helsel, L.O.; Steigerwalt, A.G.; Vega, R.; Clark, T. a.; Daneshvar, M.I.; et al. Novel *Brucella* strain (BO1) associated with a prosthetic breast implant infection. *J. Clin. Microbiol.* **2008**, *46*, 43–49, doi:10.1128/JCM.01494-07.

14.	Tiller, R. V; Gee, J.E.; Lonsway, D.R.; Gribble, S.; Bell, S.C.; Jennison, A. V; Bates, J.; Coulter, C.; Hoffmaster, A.R.; De, B.K. Identification of an unusual *Brucella* strain (BO2) from a lung biopsy in a 52 year-old patient with chronic destructive pneumonia. *BMC Microbiol.* **2010**, *10*, 23, doi:10.1186/1471-2180-10-23.

15.	Whatmore, A.M.; Davison, N.; Cloeckaert, A.; Al Dahouk, S.; Zygmunt, M.S.; Brew, S.D.; Perrett, L.L.; Koylass, M.S.; Vergnaud, G.; Quance, C.; et al. *Brucella papionis* sp. nov., isolated from baboons (*Papio* spp.). *Int. J. Syst. Evol. Microbiol.* **2014**, *64*, 4120–8, doi:10.1099/ijs.0.065482-0.

16.	Tiller, R. V.; Gee, J.E.; Frace, M.A.; Taylor, T.K.; Setubal, J.C.; Hoffmaster, A.R.; De, B.K. Characterization of novel *Brucella* strains originating from wild native rodent species in North Queensland, Australia. *Appl. Environ. Microbiol.* **2010**, *76*, 5837–5845, doi:10.1128/AEM.00620-10.

17.	Fischer, D.; Lorenz, N.; Heuser, W.; Kämpfer, P.; Scholz, H.; Lierz, M. Abscesses associated with a *Brucella inopinata*-like bacterium in a big-eyed tree frog (*Leptopelis vermiculatus*). *J. Zoo Wildl. Med.* **2012**, *45*, 625–8, doi:10.1638/2011-0005R2.1.

18.	Soler-Lloréns, P.F.; Quance, C.R.; Lawhon, S.D.; Stuber, T.P.; Edwards, J.F.; Ficht, T.A.; Robbe-Austerman, S.; O’Callaghan, D.; Keriel, A. A *Brucella* spp. Isolate from a Pac-Man Frog (*Ceratophrys ornata*) Reveals Characteristics Departing from Classical Brucellae. *Front. Cell. Infect. Microbiol.* **2016**, *6*, 116, doi:10.3389/fcimb.2016.00116.

19.	Scholz, H.C.; Revilla-Fernández, S.; Al Dahouk, S.; Hammerl, J.A.; Zygmunt, M.S.; Cloeckaert, A.; Koylass, M.; Whatmore, A.M.; Blom, J.; Vergnaud, G.; et al. *Brucella vulpis* sp. nov., isolated from mandibular lymph nodes of red foxes (*Vulpes vulpes*). *Int. J. Syst. Evol. Microbiol.* **2016**, *66*, 2090–8, doi:10.1099/ijsem.0.000998.

20.	Eisenberg, T.; Riße, K.; Schauerte, N.; Geiger, C.; Blom, J.; Scholz, H. Isolation of a novel “atypical” *Brucella* strain from a bluespotted ribbontail ray (*Taeniura lymma*). *Antonie Van Leeuwenhoek* **2017**, *110*, 221–234, doi:10.1007/s10482-016-0792-4.

21.	Al Dahouk, S.; Köhler, S.; Occhialini, A.; Jiménez de Bagüés, M.P.; Hammerl, J.A.; Eisenberg, T.; Vergnaud, G.; Cloeckaert, A.; Zygmunt, M.S.; Whatmore, A.M.; et al. *Brucella* spp. of amphibians comprise genomically diverse motile strains competent for replication in macrophages and survival in mammalian hosts. *Sci. Rep.* **2017**, *7*, 44420, doi:10.1038/srep44420.

22.	Guzmán-Verri, C.; Suárez-Esquivel, M.; Ruíz-Villalobos, N.; Michel, S.; Gonnet, M.; Campos, E.; Víquez-Ruiz, E.; Chacón-Díaz, C.; Conde-Alvarez, R.; Moriyón, I.; et al. Genetic and phenotypic characterization of the etiological agent of canine orchiepididymitis smooth *Brucella* sp. BCCN84.3. *Front. Vet. Sci.* **2019**, *6*, 175, doi:10.3389/fvets.2019.00175.

23.	Eisenberg, T.; Schlez, K.; Fawzy, A.; Völker, I.; Hechinger, S.; Curić, M.; Schauerte, N.; Geiger, C.; Blom, J.; Scholz, H.C. Expanding the host range: infection of a reptilian host (*Furcifer pardalis*) by an atypical *Brucella* strain. *Antonie van Leeuwenhoek, Int. J. Gen. Mol. Microbiol.* **2020**, *9*, doi:10.1007/s10482-020-01448-9.
